# Thermic Fever, or Sunstroke

**Published:** 1872-10

**Authors:** 


*Thermic Fever, or Sunstroke.* By H. C. Wood, Jr., M. D. Philadelphia: J. B. Lippincott & Co., 1872. Buffalo: H. H. Otis.

This essay, which received the Boylston Prize, evinces the careful, thoughtful student, and the faithful recorder of experimental research. The work is divided into four parts: Part I. Clinical History; Part II. Nature; Part III. Treatment; Part IV. Sequelæ. Part I. occupies some thirty pages of the work and cites several illustrative cases. Affections which are liable to be confounded with sunstroke are also spoken of to some extend in this portion of the work. Our author also speaks of the mistaken idea that the direct rays of the sun are necessary to produce what is called sunstroke. Part II. occupies the most space and is the most interesting portion of the work. The author here narrates a series of observations which he made as to the effect of a high temperature on the brain of living animals. His observations were made upon the muscular system, the nervous system, and the question of blood poisoning.

The conclusions which the author draws from his observations are interesting and instructive. The subjects of treatment and the sequelæ present nothing new. In speaking of the fever following sunstroke Dr. Wood in conclusion says: "I think the evidence now brought forward is sufficient to establish the frequency of meningitis following sunstroke, and that the fixed, intense headache so often complained of is probably due to such cause."

We know of no work which gives so full an exposition of this affection. The profession are under many obligations to Dr. Wood for his excellent monograph.

